# Cue-Reactive Phenomenology Mediates the Relationship Between Positive Schizotypy and Cue-Reactive Urge to Gamble in Poker-Machine Gamblers

**DOI:** 10.1007/s10899-024-10310-w

**Published:** 2024-05-09

**Authors:** Benjamin A. McTigue, Andrew C. Talk, Kylie Rice, Adam J. Rock

**Affiliations:** School of Psychology, University of New England, Armidale, NSW 2351 Australia

**Keywords:** Poker-machines, Slot machines, Gambling cue-reactivity, Urge, Schizotypy, Phenomenology

## Abstract

Although ubiquitous in numerous nightlife cultures, poker-machines present a high risk for problematic use and addiction. Previous research has demonstrated that gambling cues (e.g., flashing lights) can activate gambling urges in poker-machine gamblers. However, the processes that contribute to the maintenance of cue-reactive urges to gamble remain unclear. Consequently, the present study explored whether positive schizotypy predicted gambling urge, and whether cue-reactive altered state of awareness, cue-reactive altered time sense, and cue-reactive absorption mediated this relationship. Seventy adults aged between 19 and 68 (*M* = 48.86, *SD* = 12.82) participated in an online cue-reactivity experiment. Participants first completed the *Problem Gambling Severity Index* and the Unusual Experiences subscale of the *Short Oxford-Liverpool Inventory of Feelings and Experiences*. Subsequently, at three time points (i.e., baseline, directly after a neutral cue, and directly after a gambling cue) participants completed the Altered State of Awareness, Altered Time Sense, and Absorption subscales of the *Phenomenology of Consciousness Inventory* and a visual analogue scale measuring cue-reactive urge to gamble. It was found that positive schizotypy was significantly positively correlated with cue-reactive urge to gamble. Additionally, cue-reactive altered state of awareness, cue-reactive altered time sense, and cue-reactive absorption mediated this relationship. The theoretical, clinical and practical implications are discussed.

*Problem gambling* may be defined as gambling behaviours that consistently create negative consequences, impacting the individual, their social network, or the broader community (Ferris & Wynne, 2001). Although not deemed functionally severe enough to constitute a formal gambling disorder, problem gambling behaviours still have pronounced negative impacts (Jazaeri & Habil, 2012), which are evident across a broad range of cultural, social, and economic backgrounds (Calado & Griffiths, 2016). Problem gambling is associated with numerous biopsychosocial impacts for individuals (Fong, 2005). For example, higher rates of psychological distress have been observed, in the form of symptoms of depression (Churchill & Farrell, 2018) and anxiety (Ste-Marie et al., 2006). Detrimental financial and interpersonal impacts are also well-documented (Mathews & Volberg, 2013), and the burden of harm extends beyond the individual to the family and broader community (García-Castro et al., 2022). Therefore, it is important to understand how gambling can be an adaptive leisurely activity for some individuals, and a highly maladaptive behaviour for others.

Poker-machine gambling yields a particularly high financial cost to users and is among the most popular methods for ‘at-risk’ or problem gamblers (Livingstone, 2017). The Australian Gambling Research Centre notes that poker-machines involve salient and novel stimuli – including auditory and visual cues – that distinguish them from other gambling methods (Livingstone, 2017). There is evidence that gamblers learn to associate the sensory stimuli (e.g., flashing lights) involved in a poker-machine win with the win itself (Ashrafioun et al., 2011). This process facilitates a conditioned response, whereby repeated exposure to sensory cues increases one’s gambling urge over time, creating a *cue-reactive urge to gamble*. Similar learning models have been implicated in other forms of addiction, including drug addiction (O’Brien et al., 1998).

## Cue-Reactive Urge to Gamble

Much of the earlier problem gambling research did not consider differences across gambling modalities and the potentially unique processes involved (Dickerson, 1993). However, gambling research has increasingly become more mode-specific, with a particular focus on poker-machine gambling (Clark et al., 2016). Modern electronic poker-machines simulate a traditional mechanical device, electronically depicting spinning wheels with clicking noises that generate a series of numbers and prizes (Walker, 2004).

Although a popular part of many nightlife cultures, poker-machines present a high risk for problematic use and addiction (Livingstone, 2017). Poker-machines might present increased risk for problematic use because they provide unique sensory experiences some users find particularly enticing (Clark et al., 2016; Livingstone, 2017; Walker, 2004). Researchers have suggested poker-machine gamblers learn to associate the novel sensory cues with a win, and that, over time, mere exposure to these cues instils an urge to gamble (Ashrafioun et al., 2011; Baudinet & Blaszczynski, 2013). This notion is supported by classical conditioning theory, which states that an event or stimulus will become emotionally salient if continuously paired with an emotion-inducing stimulus (Dawson, 1973).

Sodano and Wulfert (2009) were among the first to report empirical support for cue-reactivity in the context of problem gambling. They measured self-reported urge to gamble in problem gamblers and healthy controls after presenting a series of gambling cues, including pictures of gambling scenes and sounds of poker-machines. Participants who were identified as problem gamblers showed higher increased gambling urges than healthy controls following cue exposure. Ashrafioun et al. (2011) found a similar result using gambling-related images and a written stimulus designed to elicit gambling urges. The written scenario elicited higher self-reported gambling urges, perhaps reflecting a particularly strong influence of imagination on cue-reactive urges. However, neither study included a baseline measure of gambling urge or a neutral cue (i.e., a non-gambling-related stimulus), making it difficult to isolate the impact of the gambling-related cue.

Several studies have since rectified this design limitation. For example, Tricker et al. (2015) found that gambling urge increased from viewing a neutral cue to a gambling cue in poker-machine gamblers, whilst controlling for baseline urge to gamble. The neutral stimulus was a video depicting two adult females making tea, whereas the gambling video depicted the same two adult females playing poker-machines. McKeith et al. (2017) and Dale et al. (2019) replicated this finding, thus, providing further support for a cue-reactive urge to gamble effect.

### Positive Schizotypy

Individuals with schizotypal personality traits experience cognitive, perceptual, and interpersonal disturbances akin to those seen in schizophrenia spectrum pathologies (Cohen et al., 2015). Although schizotypal personality disorder (SPD) is a specified diagnosis in the Diagnostic and Statistical Manual of Mental Disorders (DSM-5; American Psychiatric Association, 2013), schizotypal traits also present dimensionally across the general population and do not necessarily indicate pathology (Ettinger et al., 2015). Decades of research into the factor structure of trait schizotypy has supported a three-factor model (see Allen et al., 1987; Fonseca-Pedero et al., 2018). Accordingly, trait schizotypy is now understood to include positive, negative, and disorganised traits. Positive traits feature cognitive-perceptual disturbances, negative traits feature affective and interpersonal disturbances, and disorganised traits feature speech and behavioural disturbances (Arndt et al., 1991; Rossi & Daneluzzo, 2002).

The cognitive-perceptual disturbances of positive schizotypy involve unusual beliefs and perceptual experiences thematically similar to the delusions and hallucinations in schizophrenia (Straub & Kerns, 2021). Positive schizotypy traits are of particular interest in the addiction literature (Szoke et al., 2014). For example, two meta-analyses have demonstrated replicated links between cognitive-perceptual disturbances and problematic cannabis use (Carney et al., 2017; Szoke et al., 2014). Positive schizotypy has also been linked to tobacco addiction (Goff et al., 1992; Ziedonis et al., 1994), and increased alcohol consumption (Nunn et al., 2001).

Additionally, positive schizotypy is implicated in problematic internet use (Mittal et al., 2007; Schimmenti et al., 2017). For example, Mittal et al. (2007) found adolescents with SPD use social media and internet gaming significantly more regularly than healthy controls. However, it is worth noting that this research used SPD patients and, thus, investigated general schizotypal traits rather than exclusively cognitive-perceptual disturbances. More recently, Schimmenti et al. (2017) found a positive relationship between positive schizotypy and symptoms of internet gaming disorder, suggesting unusual cognitive-perceptual experiences might be seen as more acceptable and appropriately harnessed in immersive, virtual spaces. Furthermore, Brooks and Clark (2022) recently found gambling related cognitions to be associated with schizotypy and problem gambling, and based on these findings the authors hypothesised “that high trait schizotypy may be a risk factor for disordered gambling” (p. 414). To date, there is no research specifically investigating the relationship between positive schizotypy and poker-machine gambling, despite consistent links between unusual cognitive-perceptual experiences and other addictive behaviours.

### Altered Phenomenology

Following Pekala and Rock (2024), in the present study we used the term phenomenology “to simply denote first-person reports of the qualia of consciousness” (p. 105). Pekala (1991) developed a retrospective phenomenological assessment instrument referred to as the *Phenomenology of Consciousness Inventory* (PCI) to quantify alterations in phenomenology such as state of awareness, absorption, and time sense.

Following Mason et al. (2005), Rock et al. (2008) suggested that positive schizotypy may be a trait-like tendency to experience alterations in phenomenology. Indeed, Rock et al. found that participants who scored high on the cognitive-perceptual factor of schizotypy reported statistically significant alterations in phenomenology (e.g., PCI-time sense) while visualising coupled with listening to monotonous drumming relative to participants who scored low on the cognitive perceptual factor. In another study, Rock and Kambouropoulos (2009) conducted a cue-reactivity experiment and reported that altered phenomenology (i.e., PCI-altered state of awareness) mediated the relationship between the unusual experiences trait and urge to drink alcohol. Rock and Kambouropoulos reasoned that “certain individuals who are exposed to an alcohol cue may *re-experience*, rather than merely remember, the altered state of awareness (e.g., intoxication) associated with previous alcohol consumption and this may, in turn, facilitate increases in one’s urge to drink” (p. 443). This contention may also apply to poker-machine gambling and the urge to gamble. In the present study, we investigated the PCI variables of altered state of awareness, time sense, and absorption.

#### Altered State of Awareness

The PCI dimension of *altered state of awareness* (ASA) aims to capture the general subjective phenomenon of shifts in one’s state of conscious awareness (Pekala et al., 1986). A series of studies on alcohol use consistently found that ASA was positively correlated with cue-reactive alcohol urges (Kambouropoulos & Rock, 2010; Rock & Kambouropoulos, 2009). Using a similar cue-reactive paradigm but in the context of poker-machine gambling, Tricker et al. (2015) found ASA ratings significantly increased from the presentation of a neutral cue to a gambling cue, while controlling for baseline urge to gamble.

#### Altered Time Sense

The subjective phenomenon of *altered time sense* – which is a sub-dimension of the PCI dimension of altered experience – might also be implicated in gambling urges. To date, no research has examined this possible link. Paasche et al.’s (2019) systematic review on the link between impulsivity and time processing in addictive disorders showed problem gamblers likely perceive time as slower than do healthy controls, particularly when they cannot act on their gambling urges. Further, research has found that more impulsive individuals experience the passing of time as slower than those with lower levels of impulsivity (Lawrence & Standord, 1999). Additionally, the “trance-like-state” that has often been reported by problem gamblers (Jacobs, 1988) suggests these individuals might experience altered temporal awareness when engaged in gambling activities.

#### Absorption

This reported “trance-like-state” might similarly indicate absorbed attention. Qualitative research conducted by Oakes et al. (2018) found problem gamblers reported constricted attention to the outside world when preoccupied with gambling activities. They termed this state the “zone”. Murch et al. (2017) found gamblers with more disordered gambling behaviours were more likely to report feeling “completely absorbed” in electronic gambling machines. Together, this evidence demonstrates a link between absorption and problem gambling. However, further research is needed to elucidate whether absorption is implicated in the *urge* to gamble, as opposed to gambling severity or behaviours in general.

### The Present Study

There are conceptual links and evidence of empirical associations between (1) poker-machine gambling and cue-reactive gambling urges (e.g., Dale et al., 2019); (2) positive schizotypy and addictive behaviours (e.g., Schimmenti et al., 2017); (3) positive schizotypy and altered phenomenology (e.g., Rock et al., 2008); and (4) dimensions of altered phenomenology and problem gambling behaviours (e.g., Tricker et al., 2015). Clarifying the nature and direction of these relationships may help inform our understanding of problem gambling behaviours with the aim of addressing their negative impacts.

Based on the relationships identified in the preceding sections, a multiple mediation analysis was proposed in the present study to explore the relationship between positive schizotypy, cue-reactive altered state of awareness, altered time-sense and absorption, and cue-reactive gambling urge. The rationale for this model is that positive schizotypy might promote changes in awareness, time-sense, and absorption, which, in turn, might facilitate an increased cue-reactivity response to gambling stimuli. Therefore, a hypothetical causal chain is proposed, whereby the independent variable (X), positive schizotypy, creates changes in multiple mediators M_1,_ M_2,_ M_3_, cue-reactive altered state of awareness, time-sense, and absorption, which creates changes in the dependent variable (Z), cue-reactive urge to gamble; thus X $\rightarrow$  M_1,_ M_2,_ M_3_ $\rightarrow$  Z. Consequently, the following hypotheses were tested:

- H1. Urge to gamble will increase from neutral cue to gambling cue, while controlling for baseline urge to gamble.
- H2. Positive schizotypy will predict cue-reactive urge to gamble (from neutral cue to gambling cue, while controlling for baseline urge to gamble).
- H3. Cue-reactive altered state of awareness, cue-reactive altered time sense, and cue-reactive absorption mediate the relationship between positive schizotypy and cue-reactive urge to gamble (from neutral cue to gambling cue).

## Method

### Power Analysis

Given an assumed *α* of 0.05, four predictor variables, a target power of 0.80, and, in accordance with the findings of previous poker-machine gambling studies (e.g., Dale et al., 2019; McKeith et al., 2017; Tricker et al., 2015), a medium to large effect size (*f*^*2*^ = 0.2), a power analysis was conducted using G*Power (version 3.1), which stipulated that a minimum sample size of 40 participants was required for the present study.

### Participants

A total of 121 adults participated in this study. Twenty (16.59%) participants were excluded because they did not meet inclusion criteria (including 16 whose preferred gambling method was something other than electronic gambling machines or online electronic gambling machines, and four because they had not used poker-machines within the past 12 months). An additional 31 (24.80%) participants were excluded from data analyses due to incomplete responses or missing data. The final sample consisted of 70 adults (48 males, 21 females, 1 other) aged 19 to 68 years (*M* = 48.86, *SD* = 12.82). The characteristics of the current study’s sample are presented in Table 1.

**Table 1:** Characteristics of Eligible Participants

Variable	*M (SD)*	Range	*n*	%
Age	48.86(12.82)	19–68		
Sex				
Male			48	68.57
Female			21	30
Other			1	1.43
Gambling frequency				
Daily			11	15.71
2–3 days			25	35.71
Weekly			30	42.86
Every 2 weeks			4	5.71
Problem gambling				
PGSI score	18.17(5.07)	3–27		
PGSI score of 4 + *			68	97.14

*N* = 70 for the total sample

^*^Problem gambling cut-off score recommended by Nower and Blaszczynski (2010). The same number of participants (*N* = 68) met the problem gambling cut-off score (8 +) according to the PGSI (see Ferris & Wynne, 2001)

### Measures and Materials

## Problem Gambling Severity

 Participants were asked four questions about their gambling behaviours and then administered the *Problem Gambling Severity Index* (PGSI). First, participants were asked to select from a series of nine options in response to the question “What is your most preferred or common form of gambling?”. Subsequently, participants were asked to respond either *‘yes’* or *‘no’* to the question “Have you played poker-machines in the last 12 months?”. Next, participants were asked to select from seven options, ranging from ‘*daily’* to ‘*every 3 months or less’,* in response to the question “How frequently have you played poker-machines in the last 12 months?”. Finally, participants were asked to select from five options, ranging from ‘*less than 30 min’* to *‘more than 3 h’* in response to the question “How long would you spend playing the poker-machines, each time you play?”. These questions were derived from previous poker-machine gambling cue-reactivity studies (e.g., Dale et al., 2019; Tricker et al., 2015), and were used to establish eligibility for the present study. The following inclusion criteria were used: Participants were required to be at least 18 years of age, have poker-machines as their preferred gambling modality, have played poker-machines at least once per fortnight over the past 12 months, and received a score of at least one on the PGSI.

Problem gambling severity was assessed using the PGSI (Ferris & Wynne, 2001). The PGSI is comprised of nine self-report items rated on a 4-point Likert scale (0 = *never*; 1 = *sometimes*; 2 = *most of the time*; 3 = *almost always*). The scale assesses symptoms of problem gambling in non-clinical populations, and all questions relate to the previous 12-month period. The scale includes questions capturing both problem gambling *behaviours* (e.g., “how often have you borrowed money or sold anything to get money to gamble?”) and *negative consequences* (e.g., “how often have you felt guilty about the way you gamble or what happens when you gamble?”). The PGSI has good criterion validity and strong internal consistency, with Cronbach’s alphas ranging from 0.84 to 0.97 (Nower & Blaszczynski, 2010). Cronbach’s alpha in the present study was 0.87.

Given PGSI scores of eight or more indicate problem gambling with high potential for negative consequences and lack of control (Ferris & Wynne, 2001), our participant sample represented a population with highly problematic gambling behaviours. Table 1 shows only two participants did not meet the recommended problem gambling cut-off scores proposed by Nower and Blaszczynski (2010) and Ferris and Wynne (2001).

### Gambling Urge

Urge to gamble was assessed using a visual analogue scale (VAS), which has been widely used in cue-reactivity studies (e.g., Rock & Kambouropoulos, 2012; Tricker et al., 2015). The VAS was presented as a horizontal line across the width of the online page, making it relative to the screen-size on which it was viewed. Participants were asked how strong their urge to play poker-machines was, in the present moment, ranging from 0 (*no urge*) to 10 (*extreme urge*). Participants responded by sliding the point along the line.

### Video Cues

The two video cues used in this study were created by Tricker et al. (2015). The neutral cue was a three-minute video of two adult females making a cup of tea. The audio content involved quiet chatter regarding the process of making the tea. The gambling cue was a three-minute video of the same two adult females playing poker-machines in a hotel. The audio content of this video included poker machine sounds and the two women chatting about their play and wins.

### Positive Schizotypy

Positive schizotypy was measured by the ‘unusual experiences’ subscale of the *Short Oxford-Liverpool Inventory of Feelings and Experiences* (O-LIFE; Mason et al., 2005). This subscale contains 12 items on which participants must either respond ‘*yes’* or *‘no’*. The items measure perceptual aberrations, hallucinations, and magical thinking – all of which are thematically related to the positive symptoms of psychosis (i.e., positive schizotypy). Some example items are “*Have you ever thought that you had special, almost magical powers?”* and “*Can some people make you aware of them just by thinking about you?”*. The ‘unusual experiences’ subscale of the short O-LIFE has demonstrated good internal consistency (with a Cronbach’s alpha of 0.80; Mason et al., 2005). Given evidence of high attrition rates in gambling research (e.g., Dale et al., 2019), this scale was preferrable over other longer measures of positive schizotypy. Cronbach’s alpha in the present study was 0.83.

### Altered Phenomenology

Altered phenomenology was measured using the relevant dimensions or sub-dimensions of the *Phenomenology of Consciousness Inventory* (PCI; Pekala, 1991). The PCI assesses 12 major dimensions and 14 sub-dimensions of phenomenological experience across a 7-point dipole scale. Each item is presented as a pair of questions, where participants are required to select a response from ‘*neutral*’ to ‘*very strongly agree’* in either direction toward their desired response (i.e., from 0 to 6).

The **altered state of awareness (ASA)** dimension includes three pairs of questions, including “My state of consciousness was not any different or unusual from what it ordinarily is” versus “I felt that I was in an extremely different and unusual state of consciousness”. The **altered time sense** sub-dimension includes three pairs of questions, including “I felt no sense of timelessness; time flowed as I usually experienced it” versus “Time stood still; there was no movement of time at all”. The **absorption** sub-dimension includes two pairs of questions, including “I was forever distracted and unable to concentrate on anything” versus “I was able to concentrate quite well and was not distracted”. The PCI has demonstrated strong criterion validity and internal consistency (with Cronbach’s alphas ranging from 0.70 to 0.90; Pekala et al., 1991; Rock et al., 2015).

### Design and Procedure

The present study was approved by the University of New England’s Human Research Ethics Committee (approval number: HE21-144). Participants over the age of 18 who use poker-machines were sought via flyer advertisements placed at local libraries, shopping centers, and gyms in Darwin, as well as on social media (e.g., Facebook, Reddit, and Whirlpool forums). Gambling support clinics were also contacted and asked to distribute the survey information to patients and service users.

This study used a repeated-measures design including three phases, consistent with previous cue-reactivity studies (e.g., Dale et al., 2019; McKeith et al., 2017; Tricker et al., 2015). These phases were: (1) baseline assessment; (2) neutral cue presentation; and (3) gambling cue presentation. Consistent with standard cue-reactivity protocols, cue presentation was not counterbalanced because presenting the gambling cue prior to the neutral cue may result in the urge to gamble becoming a carry-over effect that contaminates neutral cue presentation (Rohsenow & Niaura, 1999).

After reading the participant information, providing consent, and completing basic demographic information (i.e., age, gender, and country of residence), participants completed the four gambling-related questions and the PGSI. Participants were then presented with the ‘unusual experiences’ subscale of the short O-LIFE. Subsequently, they were presented with the VAS (to assess baseline urge to gamble) and the three PCI sub-scales. The neutral cue was then presented, followed by the VAS (to assess current urge to gamble), and the three PCI sub-scales, to assess phenomenology pertaining to the neutral cue. The gambling cue was then presented, again, followed by the VAS and the three PCI sub-scales, this time to assess phenomenology pertaining to the gambling cue presentation. Finally, the debriefing page was presented, which included contact details for support lines.

## Results

### Descriptive Statistics

Means, standard deviations, and maximum and minimum scores for each variable included in this study are reported in Table 2.

**Table 2:** Descriptive Statistics for Variables Used in Primary Analyses

Variable	Mean	*SD*	Minimum	Maximum
Positive schizotypy	7.00	3.41	0	12
Cue-reactive gambling urge	4.19	3.08	-2	10
Cue-reactive altered state of awareness	2.70	1.55	-1.33	4.67
Cue-reactive altered time-sense	2.79	2.09	-2.00	5.33
Cue-reactive absorption	2.88	2.11	-4.50	5.00

*Note*: In the context of Table 2, cue-reactive gambling urge and the PCI variables (i.e., altered state of awareness, altered time-sense, absorption) are change variables (from neutral cue to gambling cue), not overall scores on the scale dimensions

### Data Analysis

#### Hypothesis 1.

To test the first hypothesis that urge to gamble would increase from neutral cue to gambling cue, whilst controlling for baseline urge to gamble, a one-way repeated measures analysis of covariance (ANCOVA) was performed. Baseline urge to gamble (*M* = 2.97, *SD* = 1.77) was included as a covariate. The ANCOVA consisted of one within-subjects variable with two levels (i.e., neutral cue and gambling cue). Results indicated that, after accounting for baseline gambling urge, there was a statistically significant main effect of cue, *F*(1,12) = 41.06, *p* < 0.001, partial *η*^*2*^ = 0.90. Specifically, urge to gamble increased significantly from neutral cue (*M* = 3.00, *SD* = 1.68) to gambling cue (*M* = 7.19, *SD* = 2.55).

#### Hypothesis 2.

To test the second hypothesis that positive schizotypy would predict cue-reactive urge to gamble, whilst controlling for baseline urge to gamble, a hierarchical multiple regression analysis was conducted. Firstly, an urge-change score was calculated as a measure of cue-reactive gambling urge, which consisted of the gambling cue VAS response minus the neutral cue VAS response. This variable was entered as the dependent variable, baseline gambling urge was entered as a predictor in step 1, and positive schizotypy was entered as a predictor in step 2. After controlling for baseline gambling urge, positive schizotypy predicted a statistically significant increase in gambling urge from neutral cue to gambling cue (*b* = 0.63, 95% CI [0.50, 0.76], Δ*R*^2^ = 0.44, *p* < 0.001, *f*^*2*^ = 2.23). Positive schizotypy explained 44% of the unique variance in urge to gamble from neutral cue to gambling cue, with a large effect size (Cohen, 1992).

#### Hypothesis 3.

To test the final hypothesis that cue-reactive altered state of awareness, altered time sense, and absorption would mediate the relationship between positive schizotypy and cue-reactive gambling urge, a multiple mediation analysis was conducted using model 4 of Hayes’ PROCESS macro (Field, 2013). Five thousand bootstrap iterations were used in accordance with Hayes’ recommendation (Field, 2013).

All proposed mediation variables significantly mediated the relationship between positive schizotypy and cue-reactive gambling urge. The total indirect effect was significant, *b* = 0.16, 95% CI [-0.04, 0.36]. *P*_*M*_ = 0.77, indicating the three PCI domains mediated approximately 77% of the total effect of positive schizotypy on cue-reactive gambling urge.

**Fig. 1:**
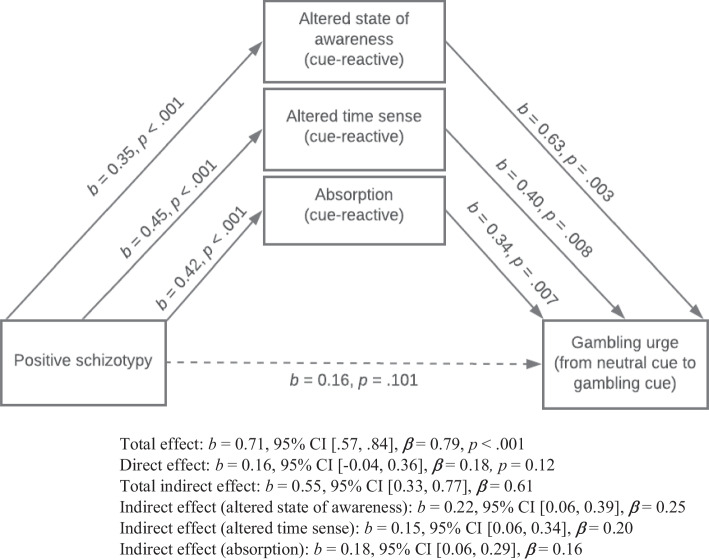
Unstandardised (*b*) and standardised (*β*) coefficients for cue-reactive altered state of awareness, altered time sense, and absorption as mediators between positive schizotypy and cue-reactive gambling urge. Non-significant pathways are denoted by dotted lines

*R*_*M*_ = 3.40, which indicates that the total indirect effect of positive schizotypy on cue-reactive urge was approximately 3.40 times the size of the direct effect. A significant indirect effect was found for positive schizotypy on cue reactive gambling urge through altered state of awareness, *b* = 0.22, 95% CI [0.06, 0.39], *P*_*M*_ = 0.31, *R*_*M*_ = 1.37. A significant indirect effect was also found for positive schizotypy on cue-reactive gambling urge through altered time sense, *b* = 0.15, 95% CI [0.06, 0.34], *P*_*M*_ = 0.25, *R*_*M*_ = 1.13. The indirect effect for positive schizotypy on cue-reactive gambling urge through absorption was also significant, *b* = 0.18, 95% CI [0.06, 0.29], *P*_*M*_ = 0.20, *R*_*M*_ = 0.89. These relationships are presented in Fig. 1.

## Discussion

The current study aimed to explore the relationship between positive schizotypy and cue-reactive urge to gamble, and whether aspects of altered phenomenology mediated this relationship. The first hypothesis that urge to gamble would increase from neutral cue to gambling cue, while controlling for baseline urge to gamble, was supported. This finding replicated past research using the same cue-reactivity paradigm (Dale et al., 2019; McKeith et al., 2017; Tricker et al., 2015). This result is also consistent with conceptualisations of classical conditioning, whereby a relevant stimulus (e.g., the flashing lights of poker machines) may become conditioned to produce the subjective experiences (e.g., urges) associated with playing poker-machines (Czerny et al., 2008).

The second hypothesis that positive schizotypy would predict cue-reactive urge to gamble, while controlling for baseline urge to gamble, was also supported. This is a novel finding, as this relationship had not previously been examined. However, this finding is consistent with the well-documented evidence that positive schizotypy is implicated in addictive behaviours involving cue-reactive processes (Nunn et al., 2001; Schimmenti et al., 2017). Support for the second hypothesis, thus, highlights that cue-reactivity might be a particularly salient route to addiction for individuals who report unusual cognitive-perceptual experiences.

The present study also found support for the third hypothesis that cue-reactive altered state of awareness, cue-reactive altered time sense, and cue-reactive absorption would mediate the relationship between positive schizotypy and cue-reactive gambling urges. Again, although these relationships have not been explored previously in the literature, these findings are consistent with theoretical and empirical evidence that aspects of altered phenomenology are linked to positive schizotypy (Rock et al., 2008) and cue-reactive urges (Tricker et al., 2015).

Altered state of awareness was the strongest mediator in the present study’s mediation model. Previous studies have supported the link between altered states of awareness and cue-reactive urges, both for alcohol (Rock & Kambouropoulos, 2012) and poker-machine gambling (Tricker et al., 2015). To be in an altered state of awareness is to experience ‘reality’ as different from one’s subjective norm (Pekala, 1991). The present study’s findings imply that individuals with positive schizotypyal traits who engage in problem gambling behaviours might do so because their urge to gamble is heightened by altered awareness in the presence of poker-machine cues. The present study’s results also suggest that altered time sense may drive the urge to gamble, perhaps because individuals with high positive schizotypy are more prone to experiencing the “trance-like-states” that have been reported in problem gamblers, which compromise one’s sense of temporal awareness (Jacobs, 1988; Paasche et al., 2019). Absorption was also found to be a significant mediator of the relationship between positive schizotypy and cue-reactive gambling urge, which extends Murch et al.’s (2017) findings that individuals with problematic gambling behaviours reported feeling completely absorbed when using electronic machines. This result also supports and extends Oakes et al.’s (2018) finding that problem gamblers demonstrate restricted external attention when engaging in gambling activities.

### Theoretical Implications and Interpretations

It has been proposed that individuals with positive schizotypyal traits are more prone to addictive behaviours as they serve a temporary symptom reduction function (e.g., Schimmenti et al., 2017). The present study’s findings appear to support this contention, with positive schizotypy producing changes in phenomenology, which, in turn, increased cue-reactive gambling urges. Individuals with higher positive schizotypy experienced more pronounced changes in their state of awareness, time sense, and degree of absorbed attention These experiences are all conceptually related to escapism within activity engagement, which has been characterised by task absorption, temporary dissociation, and reduced self-evaluation (Stenseng et al., 2012). As proposed by Schimmenti et al. (2017) in relation to online gaming, a sense of escapism may motivate individuals with high positive schizotypy to become more engaged in poker-machine play and other behaviours that provide an opportunity to escape from reality and reduce symptomology. Escapism has been extended to be a self-regulatory construct, serving as a motivation that can both promote positive affect and prevent negative affect, and reduce self-awareness (Stenseng et al., 2012). Thus, gambling may serve a coping or escape function for people with higher levels of schizotypy (Brooks & Clark, 2022). However, we acknowledge that variables other than positive schizotypy (e.g., personality traits such as transliminality, dissociation, openness to experience) may promote alterations in phenomenology.

It is also plausible that individual difference variables, such as positive schizotypy, might predispose individuals who engage in problem gambling to develop a heightened cue-reactive response in relation to altered phenomenology. This heightened cue-reactive response may be related to reward processes. Gaming machines utilize schedules of reinforcement wherein a particular behaviour is rewarded after only a small percentage of occurrences of that behaviour. Research has shown that behaviours that have been conditioned in this way are more resistant to extinction, particularly when extinction occurs after partial reinforcement schedules (known as the partial reinforcement extinction effect; Capaldi, 1966) or when lean partially reinforced schedules are used during extinction (Bouton et al., 2004; Gibbs et al., 1978). These schedules not only increase the resistance of behavioural responses to extinction, but also heighten urges compared to controls. For example, individuals undergoing extinction of responses for food rewards who receive partial reinforcement during extinction not only show resistance to extinction, but also experience a greater urge to eat (van den Akker et al., 2015).

There is some evidence that suggests individual differences in susceptibility to resistance to extinction under partially reinforced conditions may be related to differences in brain dopaminergic systems. Dopamine has been widely identified as a candidate neurotransmitter involved in problem gambling (Peters et al., 2020), and has also been linked to schizotypyal experiences and behaviours (Mohr & Ettinger, 2014). Individuals with high schizotypal personality scores showed an enhanced partial reinforcement extension effect compared to a low schizotypy group (Gray et al., 2002). Likewise, high-frequency gamblers showed increased resistance to extinction following partial reinforcement (Horsley et al., 2012). Together, these findings suggest potential neural and cognitive links between schizotypy, gambling, and gambling cue-reactivity.

### Practical and Clinical Implications

As this is the first known study to examine both positive schizotypy and aspects of altered phenomenology in relation to poker-machine gambling, these findings uniquely contribute to our understanding of the urge to gamble, and, by extension, problem gambling behaviours. The altered phenomenology variables explored in the present study are not currently actively addressed in interventions for problem gambling behaviours. Thus, treatment for problem gambling might benefit from assessing and targeting cue-reactive altered state of awareness, altered time sense, and/or absorption. By assessing cue-reactive altered states, practitioners could gain insight into the severity and types of altered states that are present for individual clients, and assess the function of these states within the idiographic gambling maintenance cycle. Furthermore, altered phenomenology may prove to be a useful cognitive target for problem-gambling treatment, to disrupt the temporary escapism within activity engagement, by reducing task absorption, disrupting temporary dissociation, and increasing self-evaluation (Stenseng et al., 2012). While the function and consequences of these altered phenomenological effects warrant investigation, it is likely that prolonged heightened states are associated with a physiological stress-response in gamblers, and may play a role in problematic use and relapse (Fong, 2005). A heightened cue-reactive response in relation to altered phenomenology may be related to reward pathways for the gambling behaviour, and extinction of this conditioning may serve to enhance treatment effects. Furthermore, identifying cue-reactivity to stimuli may have utility as a marker for disorder severity and monitoring response to treatment, as well as risk of relapse (García-Castro et al., 2022).

Additionally, whilst high positive schizotypy traits have been linked to increased likelihood of engaging in addictive behaviours (e.g., Schimmenti et al., 2017), the present study is the first to make this link, experimentally, in the context of the cue-reactivity paradigm and gambling urge. This finding has implications for clinician’s working with individuals with high positive schizotypy (e.g., in individuals with SPD and other schizophrenia spectrum disorders). Firstly, given the results of this study and evidence for the co-morbidity of schizotypy and problematic gambling behaviour (Brooks & Clark, 2022), clinicians should screen individuals with elevated schizotypy for gambling activities. For clients identified to have co-morbid schizotypy and problematic gambling, assessment of cue-reactive altered states and gambling urge may be indicated, with the goal of identifying cue-reactive responses to specific addictive stimuli for targeted intervention. For example, cue-specific response patterns could be specifically targeted in treatment with extinction mechanisms (Wulfert et al., 2009).

The overwhelming majority (97.14%) of participants in the present study had PGSI scores indicating strongly problematic use (Ferris & Wynne, 2001). Most of the data was collected from the Northern Territory in Australia, which has been found to have the highest per capita gambling expenditure nationwide, as well as the highest poker machine and betting participation rates (Menzies School of Health Research, 2018). While the high proportion of participants with scores indicative of problematic gambling may be, at least partly, explained by these statistics, the findings also draw attention to the level of problematic gambling that may be present in the general population. While problematic gambling behaviours are often sub-clinical and do not meet the full diagnosis for a gambling disorder, these behaviours are associated with numerous harmful biopsychosocial impacts for individuals, families and communities (Fong, 2005). The proportion of participants in this general population study who meet the cut-off for problematic use implicates healthcare services and clinicians to routinely screen and assess gambling behaviours more widely, to ensure that problematic use is identified and targeted.

### Limitations and Future Directions

The present study has various limitations that need to be acknowledged. The sample consisted of volunteers responding to social media and print advertisements. Therefore, it is unclear whether the characteristics and responses of the present volunteer sample accurately represented a population of poker-machine gamblers. Indeed, volunteers have been found to differ from non-volunteers across a variety of characteristics (Rosenthal & Rosnow, 1991). Furthermore, although attrition rates in the present study were not as high as those reported in previous poker-machine gambling studies (e.g., Dale et al., 2019), meta-analytic research shows problem gambling is associated with impulsivity (Ioannidis et al., 2019), meaning participants may have been less likely to engage with the neutral and gambling cues relative to non-problem gamblers.

It is also noteworthy that, as previously discussed, the majority of the data collected in this study came from one geographical region in the Northern Territory, Australia. Future research should collect data more widely, utilizing online platforms, to ensure findings are generalizable to a wider population of interest. Finally, as noted by other researchers using similar study designs (Dale et al., 2019; McKeith et al., 2017), the ecological validity of the gambling cue should be considered. The video representation of poker-machine gambling might have produced less prominent or different effects than the real-world environments in which poker-machines are used. Nonetheless, the significant results found in this study suggest the strength of the relationships examined are perhaps prominent enough to overcome such design limitations. It would be beneficial to investigate the interplay between positive schizotypy, altered phenomenology, and cue-reactive gambling urges using an in vivo or real-world context while still acknowledging the limitations inherent in these designs.

## Conclusion

The present study was the first to demonstrate a link between positive schizotypy and cue-reactive urge to gamble, with results indicating cue-reactive altered state of awareness, altered time sense, and absorption mediated this relationship. These novel findings emphasise the value in examining altered phenomenology in the context of exposure-type therapies for problematic levels of poker-machine gambling. Altered phenomenology may be a cognitive factor that serves to maintain problematic gambling behaviour. Consequently, screening and assessing for cue-reactive altered phenomenology with individuals with positive schizotypyal traits and gambling tendencies may provide additional treatment targets. Once identified, idiographic cue-specific response patterns may be targeted with extinction mechanisms (Wulfert et al., 2009), which may serve to reduce the urge to gamble. Whereas previous research has neglected to explore the impacts of both positive schizotypy and altered phenomenology on cue-reactive addictive behaviours, the present study indicates both constructs are uniquely and interdependently implicated in poker machine gamblers’ cue-reactive gambling urges.

## Data Availability

The datasets analysed during the current study are available from the corresponding author on reasonable request.
